# First principles study of double perovskites Li_2_AgAsX_6_ (X = Cl, Br, I) for optoelectronic and thermoelectric applications

**DOI:** 10.1039/d4ra07969h

**Published:** 2025-02-17

**Authors:** Ghulam M. Mustafa, Bisma Younas, Ahmad Ayyaz, A. I. Aljameel, Saud Alotaibi, S. Bouzgarrou, Syed Kashif Ali, Q. Mahmood, Imed Boukhris, M. S. Al-Buriahi

**Affiliations:** a Department of Physics, Division of Science and Technology, University of Education Lahore Punjab 54770 Pakistan; b Department of Physics, The University of Lahore Lahore Pakistan bismayounas55@gmail.com; c Centre for Advanced Studies in Physics, GC University Lahore 54000 Pakistan; d Department of Physics, College of Science, Imam Mohammad Ibn Saud Islamic University (IMSIU) Riyadh 11623 Saudi Arabia; e Physics Department, Faculty of Science and Humanities in Ad-Dawadmi, Shaqra University Shaqra 11911 Saudi Arabia Saud@su.edu.sa; f Department of Physics, College of Science, Qassim University P. O. 64 Buraidah Saudi Arabia; g Laboratoire de Microélectronique et Instrumentation (UR03/13-04), Faculté des Sciences de Monastir Avenue de l’Environnement 5000 Monastir Tunisia; h Department of Physical Sciences, Chemistry Division, College of Science, Jazan University P.O. Box. 114 Jazan 45142 Saudi Arabia; i Department of Physics, College of Science, Imam Abdulrahman Bin Faisal University P. O. Box 1982 Dammam 31441 Saudi Arabia; j Basic and Applied Scientific Research Center, Imam Abdulrahman Bin Faisal University P. O. Box 1982 Dammam 31441 Saudi Arabia; k Department of Physics, Faculty of Science, King Khalid University P. O. Box 9004 Abha Saudi Arabia; l Department of Physics, Sakarya University Sakarya Turkey

## Abstract

The present communication aims to provide a theoretical examination of the structural, electronic, optical, transport, and mechanical characteristics of Li_2_AgAsX_6_ (X = Cl, Br, I) to check their potential for optoelectronic and thermoelectric applications. The structural analysis reveals their cubic symmetry, and their structural and thermodynamic stability is verified through assessments of their tolerance factors (0.96, 0.94, and 0.93) and formation energies (−3.63, −3.10, and −2.16 eV). Their mechanical stability and ductile nature are confirmed using elastic constants, Poisson's ratio, and Pugh's criterion. Analysis of the band structure exhibits bandgaps of 0.86, 0.56, and 0.22 eV for Cl-, Br- and I-based compositions. Analysis of their optical behavior is carried out in terms of complex dielectric constant, complex refractive index, optical conductivity, reflectivity, and loss, providing better insight into material characteristics. The highest absorption in the infrared region underscores their prospects as infrared detectors. Additionally, the materials exhibit high electrical conductivity, and ultra-low lattice thermal conductivity with a considerable figure of merit, highlighting their feasibility for thermoelectric devices.

## Abstract 2

This study aims to provide a theoretical examination of the structural, electronic, optical, transport, and mechanical characteristics of Li_2_AgAsX_6_ (X = Cl, Br, I) to check their potential for optoelectronic and thermoelectric applications.

## Graphical abstract

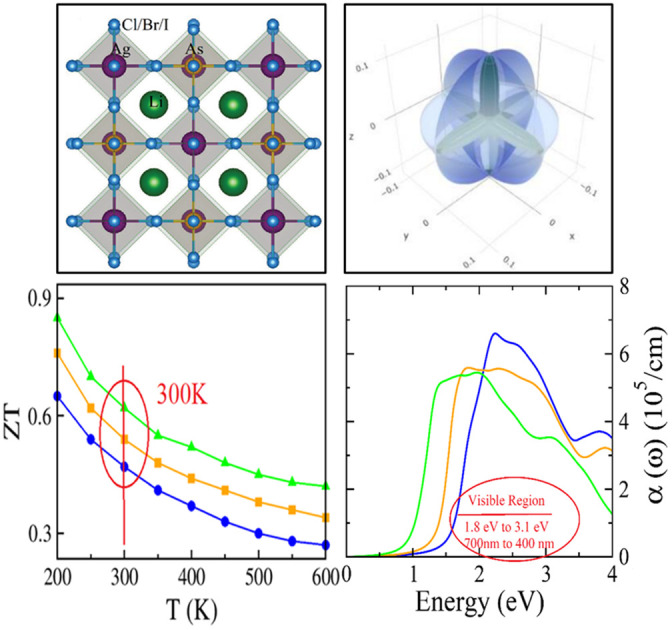

## 1. Introduction

Optoelectronic devices have the potential to transform different aspects of civilization and modern industry by improving energy production, healthcare, and scientific research.^1^ Their ability to manipulate light with high precision is a gateway to innovation and revolution in different fields. Optoelectronics is key to advancing renewable energy solutions and storage systems. Optoelectronic devices in the IR range represent the most crucial element. This spectral domain includes wavelengths longer than visible light and is useful in various applications due to its properties and abilities.^2^ Furthermore, IR optoelectronic devices are good candidates for new applications, for instance, photonics and quantum computing, which will stimulate the creation of new technologies and products and the development of technologies based on IR spectroscopy. Overall, the technical advances signify that the role and application of IR optoelectronic devices will increasingly find their niche, establish new trends, and pave a brand-new path in the future. In IR optoelectronics, double perovskite materials showed signs of stimulation and potentiality.^3,4^ These materials have great potential to enhance the efficiency and operation of devices working in the IR region of the electromagnetic spectrum because of their unique band structure and optoelectronic properties.^5^

Double perovskite materials are highly flexible in terms of their structures and they possess several interesting characteristics, suggesting they may be promising candidates for use in optoelectronics.^6^ These compounds have the formula A_2_BB′X_6_, where A is often a large cation, B and B′ are various transition metal cations, and X is an anion that may be oxygen or a halogen. These materials have amazing structural characteristics, which are correlatively connected with different physical characteristics. They can vary their behavior due to their chemical nature, allowing researchers to adapt their characteristics to fit the intended purpose. The optical properties of double perovskites revolve around their flexibility in capturing one of the most stimulating elements.^7^ These materials exhibit a wide range of optical properties, including photoluminescence, absorption, and emission, that can be modified by doping level, composition, or crystal structure.^8,9^ The optical properties of these materials are a key starting point for advancing new multi-functional optoelectronic applications, including photodetectors, solar cells, and light-emitting diodes (LEDs). In addition, double perovskites show remarkable thermoelectric performance, enabling them to efficiently convert heat into electrical power.^10^ To be a good thermoelectric contender, a material must have low thermal conductivity, high electrical conductivity, and a high Seebeck coefficient.^11^ Due to these materials having such characteristics, they can be used in waste heat recovery, solid-state cooling, and power generation, which is pivotal in the search for a sustainable energy source.^12^

Researchers are investing significant time studying the prospects of using double perovskites in optoelectronics because of their adjustable bandgap properties and prospects for application in LEDs, photodetectors, and solar cells. These materials represent several opportunities for the development of optoelectronics, as they present unique behavior to solve the challenges related to efficient light emission, absorption, and energy conversion. For instance, Abbas *et al.* studied Li_2_AgGaX_6_ (X = Cl, Br, and I) double perovskites, focusing on their mechanical, optical, and transport properties, as characterized by first-principles calculations. Their work offered detailed investigations of the materials and their interactions with the environment, enhancing the understanding of several potential applications.^13^ In addition, Zafar *et al.* used DFT to analyze the transport and optoelectrical properties of K_2_NaTlX_6_ (X= Cl, Br, I) compounds for renewable energy applications. The results have provided useful information to create an integrated platform for advancing renewable energy technologies.^14^ Rehman *et al.* used DFT to analyze the structural, optoelectronic, mechanical, and thermal properties of Cs_2_AgBiX_6_ (X = Cl, Br, I) DPs for photovoltaic application. They conducted extensive studies on these materials, highlighting their inherent characteristics and ability to convert energy from sunlight. Their findings presented important information for designing and optimizing photovoltaic devices.^15^ Likewise, Iqbal *et al.* conducted DFT-based computations to observe the electrical and thermal attributes of K_2_AgSbX_6_ (X = Cl, Br) and explored their capacity for solar cell applications. Their research was fundamental for unraveling the effect of the material's behavior when subjected to different compositions, thereby providing a basis for improving solar energy conversion efficiency. The results obtained have given an excellent indication of ways to improve high-efficiency and stable solar cells with K_2_AgSbX_6_ materials.^16^ In their research paper, Soni *et al.* studied Cs_2_ZSbX_6_ (Z = Ag, Cu and X = Cl, Br, I) as possible candidates for photovoltaic devices, emphasizing their mechanical stability and light absorption properties. Their examination showed that the most prominent feature of these materials is their pronounced mechanical resistance and huge light absorption value, which establish them as prospective photovoltaic tuning samples. Their results were the building blocks for factors such as efficiency and durability for designing and developing solar energy harvesting devices.^17^

The current report holds enormous significance for developing optoelectronics technology, using density functional theory (DFT) to comprehensively explore the structural, electronic, optical, thermoelectric, and mechanical parameters of Li_2_AgAsX_6_ (X = Cl, Br, I) double perovskites. Integrating theoretical approximations offers significant understanding of key mechanisms leading to the performance of these materials in several optoelectronic device applications. Such holistic insight is crucial for the rational design and optimization of next-generation devices with improved functionality and efficiency.

## 2. Computational methodology

In the current study, we applied the density functional theory (DFT)-centered FP-LAPW process available in WIEN2k to investigate the electronic and transport response of Li_2_AgAsX_6_ (X = Cl, Br, I).^18–20^ Initially, lattice parameters and bulk moduli were optimized before examining the band structure with the PBEsol approximation. This approximation calculates the structural properties correctly, but fails to evaluate the bandgap accurately. Therefore, for the accuracy of the bandgap, the most versatile approach of TB-mBJ has been adopted. The energy released during optimization was applied to the Murnaghan equation of state to ascertain their ground-state properties, employing the PBEsol approach.^21^ Initially calculated using the mBJ potential, the optical properties were further refined using the Trans-Blaha (TB-mBJ) extension.^22,23^ During the initialization process, *R*_MT_ × *K*_max_ and *G*_max_ were set to 8 and 12, respectively, with a *K*-mesh of 20 × 20 × 20. Energy convergence criteria were established at 10^−4^ Ry for self-consistent field computation. The optical response was calculated using the Kramer–Kronig expression,^24^ while thermoelectric properties were evaluated with BoltzTraP code.^25^

## 3. Results and discussion

### 3.1. Structural properties

**Fig. 1:**
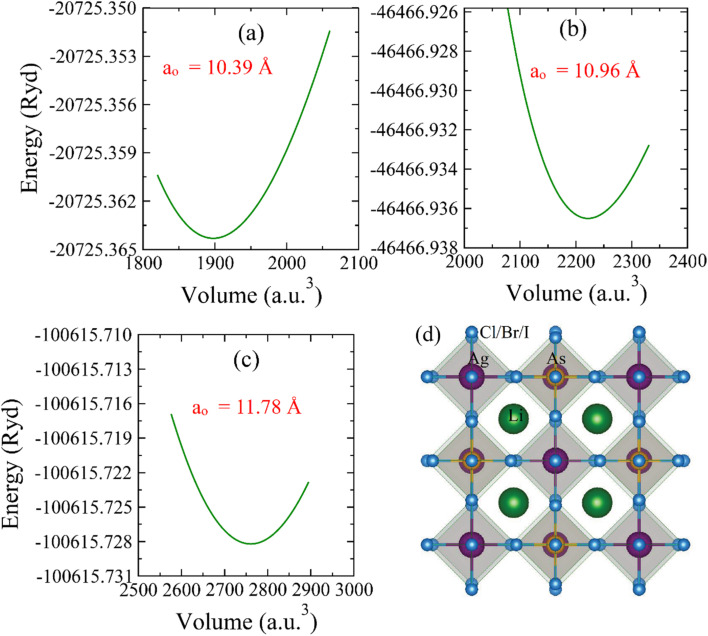
Energy–volume plots for (a) Li_2_AgAsCl_6_, (b) Li_2_AgAsBr_6_, (c) Li_2_AgAsI_6_. (d) Cubic unit cell of Li_2_AgAsX_6_ (X = Cl, Br, I).

To measure the ground-state parameters and ground-state energy, the crystal structures of these materials were optimized, which belong to the *Fm*3̄*m* space-group (no. 225).^26^ The optimization graphs for all these compositions are presented in Fig. 1(a–c). Each unit cell comprises ten atoms, and the structural parameters were determined by optimizing the geometry using the GGA method proposed by Perdew, Burke, and Ernzerhof.^27^ This optimization involves minimizing the unit cell energy relative to its volume. The figure demonstrates that increasing the volume to a certain value reduces the unit cell energy. Beyond this volume, any further increase in volume results in higher energy, indicating instability.^28^ The unit cell's most stable state, corresponding to minimum energy, is used for calculating the ground-state lattice parameter (*a*_o_) and bulk modulus (*B*_o_), which are listed in Table 1.^29^Table 1 shows that *a*_o_ increases when X is replaced by Cl to I in Li_2_AgAsX_6_, probably because of the increasing atomic radius of X atoms from Cl to I. Similarly, *B*_o_ reduces from Cl to I because of an inverse relationship between *a*_o_ and *B*_o_. The unit cell of Li_2_AgAsX_6_ is presented in Fig. 1(d). For the thermodynamic analysis, the energy of formation (Δ*H*_f_) was computed by utilizing the following equation:

1 Δ*H*_f_ = *E*_(Li_*a*_Ag_*b*_As_*c*_X_*d*_)_ − *aE*_Li_ − *bE*_Ag_ − *cE*_As_ − *dE*_X_

where *E*_(Li_*a*_Ag_*b*_As_*c*_X_*d*_)_ represents the total energy of the Li_2_AgAsX_6_ compound, and *E*_Li_, *E*_Ag_, *E*_As_, and *E*_X_ are the energies of the individual atoms.^30^ The computed Δ*H*_f_ values, presented in Table 1, are negative, showing that the investigated double perovskites are thermodynamically stable. Goldsmith's tolerance factor (*t*_G_) is used to evaluate the structural stability of a material. It is computed with the formula:2
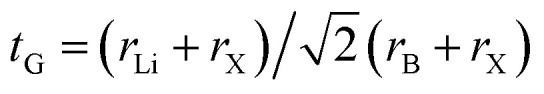
where *r*_Li_, *r*_B_, and *r*_X_ are the ionic radii of Li, Ag, As, and X, respectively. *r*_B_ is the average radius of Ag and As atoms. According to Goldsmith's criterion, for ideal cubic perovskites, the value of the Goldsmith tolerance factor (*t*_F_) lies within the range 0.8–1.4.^31^ The computed values of *t*_F_ in Table 1 confirmed the structural stability of all these compositions.

**Table 1:** The computed structural and optical features for Li_2_AgAsX_6_ (X = Cl, Br, I)

Parameter	Li_2_AgAsCl_6_	Li_2_AgAsBr_6_	Li_2_AgAsI_6_
*a*_o_ (Å)	10.40	10.96	11.78
*B* (GPa)	33.10	24.99	25.40
Δ*H* (eV)	−3.63	−3.10	−2.16
*t*_F_	0.96	0.94	0.93
*E*_g_ (eV)	0.86	0.56	0.22
*ε*_1_(0)	7.50	9.00	13.12
*n*(0)	2.73	3.00	3.63
*R*(0)	0.216	0.250	0.323

### 3.2. Electronic properties

**Fig. 2:**
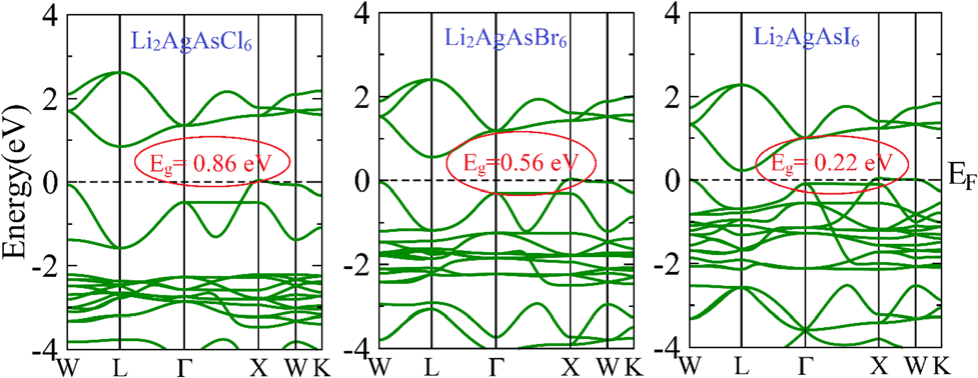
Computed band structure for Li_2_AgAsX_6_ (X = Cl, Br, I).

**Fig. 3:**
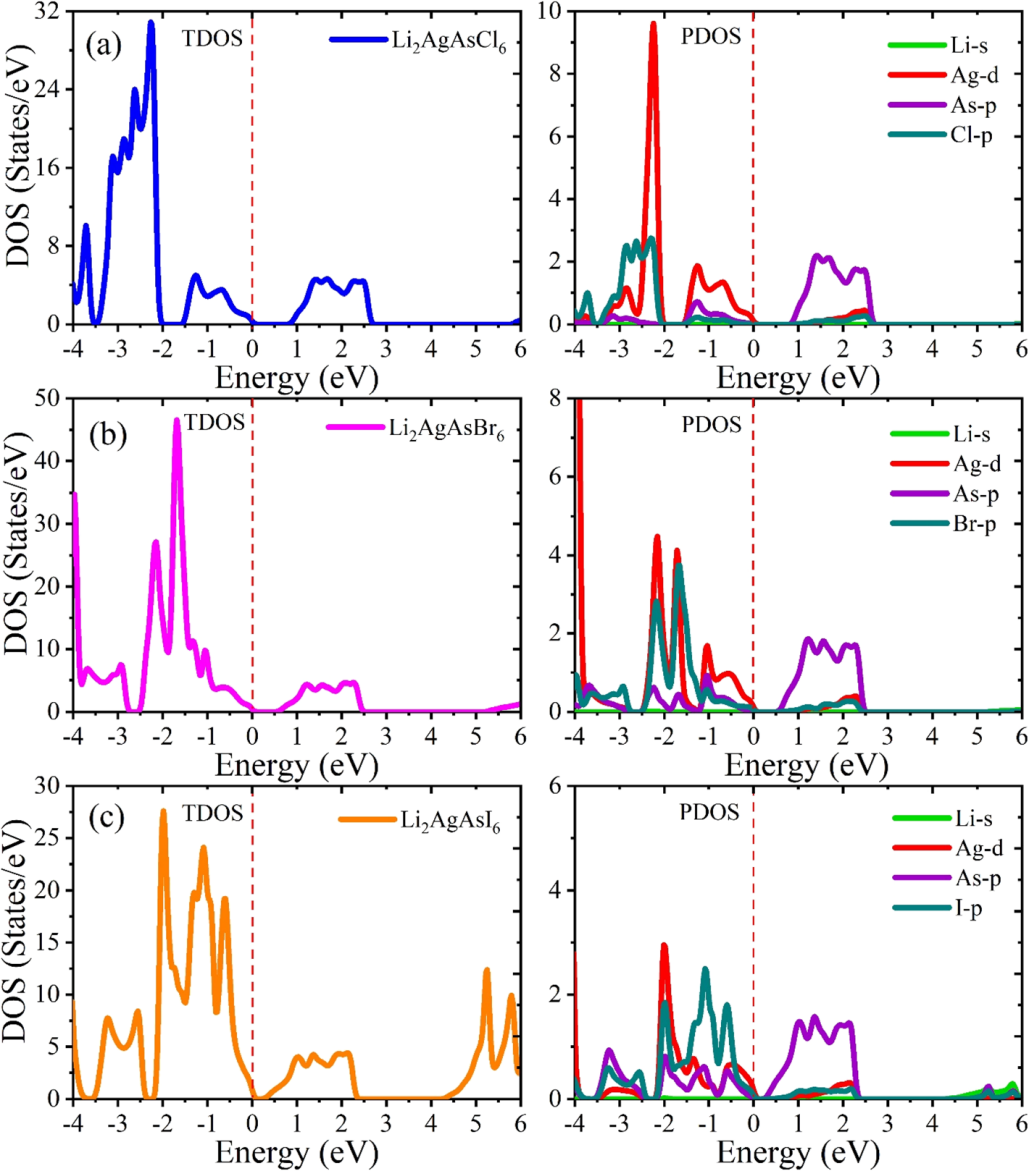
Calculated total (TDOS) and partial (PDOS) density of states for Li_2_AgAsX_6_ (X = Cl, Br, I).

The electronic properties of the investigated compositions are analyzed through their band structures to determine the nature and value of their bandgaps and density of states (DOS) to identify their potential for electronic transitions.^32^ The computed band structures are shown in Fig. 2. It is observed that the edge of the valence band (VB) is positioned at the X point. In contrast, the edge of the conduction band (CB) lies at the L symmetry point, indicating the indirect bandgap nature of these semiconductors. The bandgap value for Li_2_AgAsCl_6_ is seen to be 0.86 eV, decreasing to 0.56 eV for Li_2_AgAsBr_6_ and 0.22 eV for Li_2_AgAsI_6_.^33^ This reduction in bandgap value can be understood based on an increase in atomic size, a decrease in electronegativity, a reduction in orbital overlap, and strong spin–orbit coupling. The small bandgap values exhibit the potential of these compositions in optoelectronic devices operating in the infrared region. The density of states (DOS) plots provide insight into the role of various elemental states in band formation and bandgap tuning. Near the Fermi level (*E*_F_), the formation of VB maxima involves primarily the d-state of Ag and the p-states of As and halides.^34^ In this band, the highest density of states is that of the d-states of Ag. Conversely, CB minima result from the hybridization of the d-states of Ag and the p-states of As and halogens, with the dominant contribution from the p-states of As. It is also evident from the PDOS spectra that the substitution of larger-sized halogen atoms pushes the CB minima toward the Fermi level, leading to a reduction in the bandgap of Br and I-based compositions.^35^ This decrease in bandgap is attributed to the increased covalent character of halide atoms surrounding B-site atoms (Fig. 3).

### 3.3. Optical properties

**Fig. 4:**
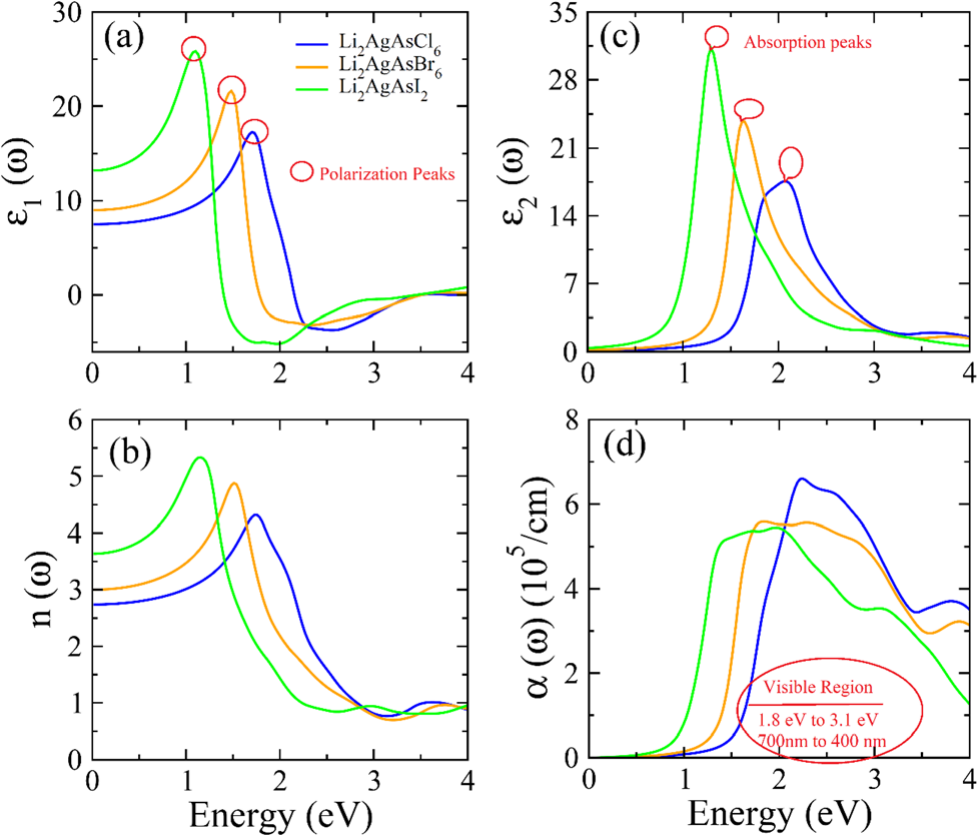
The computed (a) *ε*_1_(*ω*), (b) *ε*_2_(*ω*), (c) *n*(*ω*) and (d) *α*(*ω*) for Li_2_AgAsX_6_ (X = Cl, Br, I).

**Fig. 5:**
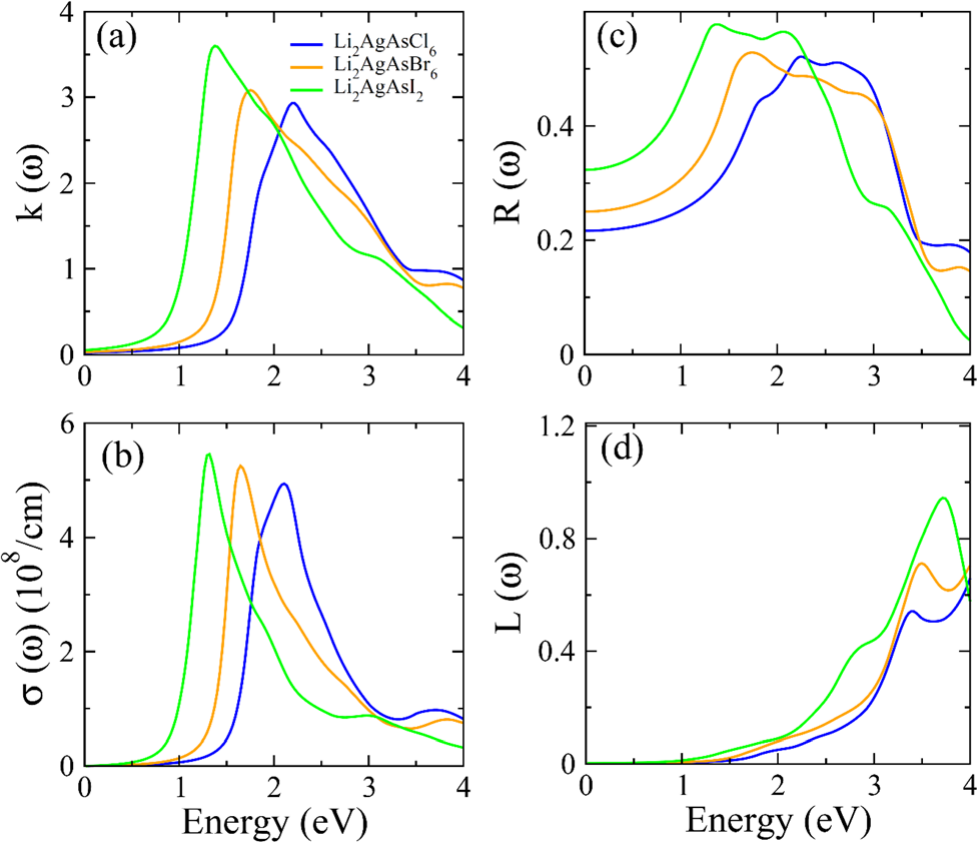
The computed (a) *k*(*ω*), (b) *σ*(*ω*), (c) *R*(*ω*), (d) *L*(*ω*) for Li_2_AgAsX_6_ (X = Cl, Br, I).

The dielectric characteristics of Li_2_AgAsX_6_ (X = Cl, Br, I) are analyzed using the complex dielectric constant *ε*(*ω*) = *ε*_1_(*ω*) + *iε*_2_(*ω*) and related features. The real *ε*_1_(*ω*) and imaginary *ε*_2_(*ω*) components of these parameters probe the polarization of the material and dispersion of incident light during the interaction of light with the material.^36^ The computed values of all optical parameters are plotted against the energy of the incident light in Fig. 4 and 5. In Fig. 4(a), the value of *ε*_1_(*ω*) is plotted against the energy of incident photons, showcasing peak values at 1.73 eV for Li_2_AgAsCl_6_, 1.50 eV for Li_2_AgAsBr_6_ and 1.24 eV for Li_2_AgAsI_6_. The positions of these peaks are determined by the resonance frequency of the relaxation phenomenon present in the material. The shifting of peaks towards lower frequency with the increasing size of the halogens shows that heavier materials move slowly and have a smaller resonance frequency. In contrast, the peak height increased with an increase in the size of the halogen, which is attributed to the greater polarizability of larger-sized halogens. The static value of the real part of the dielectric constant *ε*_1_(0) is linked to the optical bandgap (*E*_g_) *via* Penn's model, where *ε*(0) ≈ (ℏ*ω*_p_/*E*_g_)^2^.^37^ Furthermore, trends observed in *ε*_1_(*ω*) mirror those in *n*(*ω*), indicating similar dispersive behavior and material transparency, as depicted in Fig. 4(b). The static *ε*_1_(0) and *n*(0) values are connected through the expression 
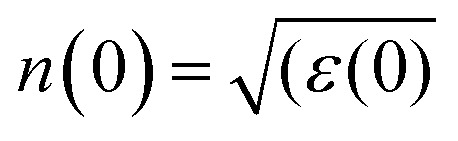
, as illustrated in Table 1.^38^ The peaks of *n*(*ω*) appear for Li_2_AgAsCl_6_ at 1.81 eV, for Li_2_AgAsBr_6_ at 1.50 eV, and for Li_2_AgAsI_6_ at 1.15 eV, signifying their importance in optoelectronic applications. The absorption attributes of a material are delineated by *ε*_2_(*ω*), as graphed in Fig. 4(c). The determination of *E*_g_ is facilitated by identifying the threshold values of *ε*_2_(*ω*). Notably, absorption bands within the ranges of 1.61–2.51 eV for Li_2_AgAsCl_6_, 1.46–2.30 eV for Li_2_AgAsBr_6_, and 1.0–2.1 eV for Li_2_AgAsI_6_ confirm light absorption in the infrared spectra. Substituting Cl with Br and I induce a shift in absorption bands towards lower energies, leading to a red shift from the visible to the infrared region.^39^ Additionally, a reduction in light energy is quantified by the absorption coefficient *α*(*ω*), as portrayed in Fig. 4(d). The absorption profile provides a wealth of information regarding the electronic and optical responses of a material and serves as a decisive parameter from the perspective of applications.^40^

Fig. 5(a) presents a graph of the energy-dependent extinction coefficient *k*(*ω*), highlighting the ratio of maximum to minimum transmission energy at specific energy levels. This graph mirrors the behavior of *ε*_2_(*ω*) due to their connection through the Kramer–Kronig relation. Optical conductivity *σ*(*ω*), shown in Fig. 5(b), represents the electron flow within optical materials as a result of light–matter interactions.^41^ The pattern of *σ*(*ω*) aligns with that of *α*(*ω*) because greater absorption of energy leads to more excited electrons, leading to increased *σ*(*ω*). The plot of reflectivity *R*(*ω*), depicted in Fig. 5(c), reveals surface characteristics. According to Table 1, *R*(*ω*) values at zero energy rise when Cl is substituted by Br or I, which is attributed to their greater size and the higher number of Br and I electrons than for Cl. This results in enhanced reflectivity in the visible spectrum, indicating the compound's suitability for optoelectronic applications.^42^ Some energy is lost during light–material interactions through scattering, heating, and thermal agitation. This energy loss is represented by the loss function *L*(*ω*), illustrated in Fig. 5(d). Comprehensive analysis of the optical spectrum confirms that these materials exhibit maximum absorption and minimal energy loss in the visible region, making the investigated compositions highly promising for renewable energy devices.^43^

### 3.4. Thermoelectric properties

**Fig. 6:**
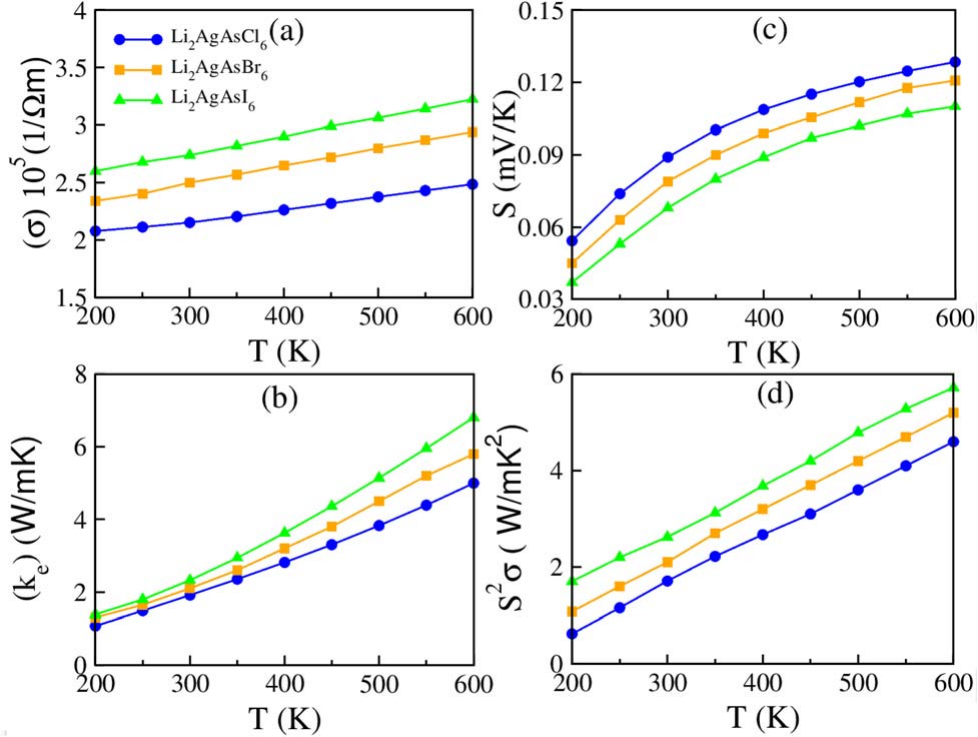
The computed (a) *σ*, (b) *k*_e_, (c) *S*, (d) PF for Li_2_AgAsX_6_ (X = Cl, Br, I).

One critical parameter for assessing a material's energy harvesting capabilities is its suitable bandgap, essential in energy conversion devices. A thermoelectric material has attracted significant attention in the past due to its capability to convert waste heat into electrical energy.^44^ The thermoelectric characteristics of materials can be determined by utilizing the BoltzTraP code within WIEN2k software. The properties involving electrical conductivity (*σ*), thermal conductivity (*κ*_e_), Seebeck coefficient (*S*), power factor (PF), specific heat capacity (*C*_v_), *κ*_e_/*σ* ratio, lattice thermal conductivity (*κ*_L_), and figure of merit (*ZT*) are elaborated with BoltzTraP code. The relaxation time typically ranges between 10^−13^ and 10^−14^ s for most semiconductor materials. In this study, a value of 10^−14^ s is used. For an effective thermoelectric material, high values of *σ* and *S* and low values of *κ*_e_ are desirable.^45^Fig. 6(a) illustrates the variation in electrical conductivity with temperature, showing a linear increase from 2.08/2.41/2.59 × 10^5^ at 200 K to 2.50/2.92/3.25 × 10^5^ (Ω m)^−1^ at 600 K for Li_2_AgAsX_6_ (X = Cl, Br, I). The increase in temperature provides more carriers in the CB, enhancing *σ*. Notably, Li_2_AgAsCl_6_ exhibits lower *σ* values than Li_2_AgAsBr_6_ or Li_2_AgAsI_6_ due to the smaller ionic radius and higher electronegativity of Cl, leading to stronger interactions and less mobility for charge carriers. Thermal conduction occurs through two modes: one is electrons and the other is phonons, represented by the relation *κ* = *κ*_e_ + *κ*_ph_, where *κ*_ph_ and *κ*_e_ are the phononic and electronic thermal conductivities, respectively.^46^ Since BoltzTraP does not account for lattice vibration, only the electronic component of *κ*_e_ is considered in the present report. As demonstrated in Fig. 6(b), there is a rise in thermal conductivity (*κ*_e_) with temperature. At 600 K, the observed *κ*_e_ values are 5.1 for Li_2_AgAsCl_6_, 5.95 for Li_2_AgAsBr_6_, and 6.92 W m^−1^ K^−1^ for Li_2_AgAsI_6_. The value of *κ*_e_ is smaller for Li_2_AgAsCl_6_ than for Li_2_AgAsBr_6_ or Li_2_AgAsI_6_, which is likely due to the stronger phonon scattering caused by the smaller ionic radius of Cl ions, which limits the enhancement in thermal conductivity.^47^ The thermal-to-electrical conductivity ratio, *κ*_e_/*σ*, remains small at 10^−5^. Consequently, our calculated parameters indicate that the investigated compounds suit thermoelectric applications. The Seebeck coefficient (*S*) gauges potential differences relative to the temperature variance among connections between two different metals. Its plot is portrayed in Fig. 6(c). A positive value of *S* indicates that holes constitute the majority carriers, characterizing the studied materials as p-type semiconductors.^48^ At 600 K, the observed *S* values are 0.129, 0.120, and 0.112 for Li_2_AgAsCl_6_, Li_2_AgAsBr_6_, and Li_2_AgAsI_6_, respectively. The higher *S* of Li_2_AgAsCl_6_ compared to Li_2_AgAsBr_6_ or Li_2_AgAsI_6_ is attributed to the higher carrier concentration and lower electrical conductivity due to the smaller ionic radius and higher electronegativity of Cl, leading to enhanced thermoelectric performance. The power factor (PF) determines the performance of thermoelectric devices, and is reliant on *σ* and *S*^2^, as depicted in Fig. 6(d) with varying temperature. The PF values steadily rise from 200 K and reach peak values of 4.7/5.2/5.8 W m^−1^ K^−2^ for Li_2_AgAsCl_6_, Li_2_AgAsBr_6_, and Li_2_AgAsI_6_, respectively, at 600 K, showcasing the significance of these materials for thermoelectric devices.^49^

**Fig. 7:**
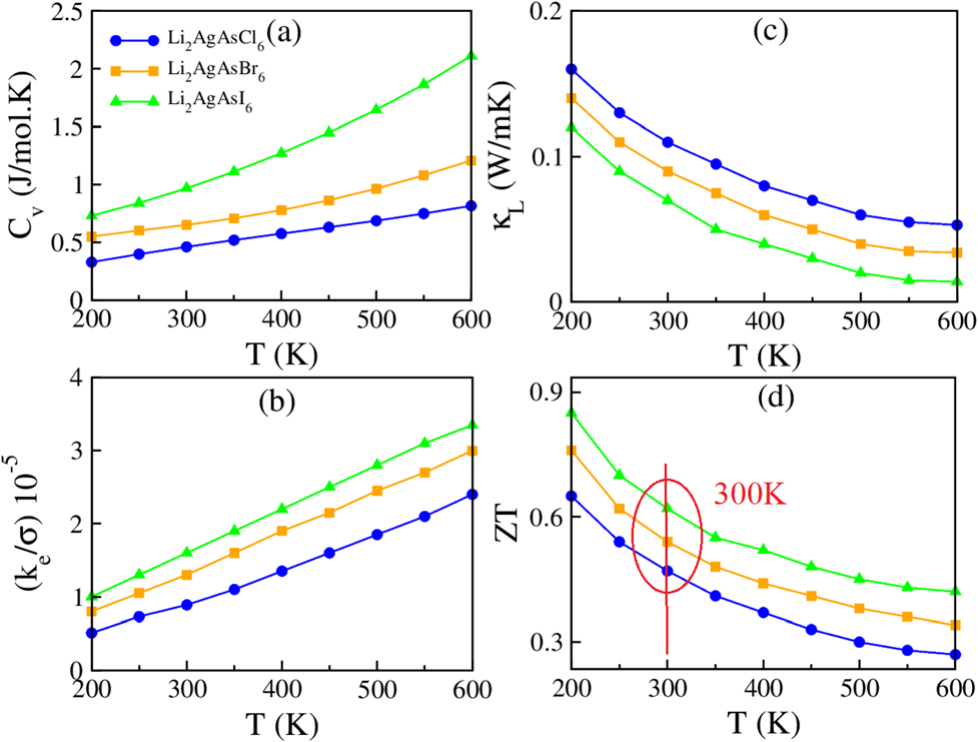
The computed (a) *C*_v_, (b) *k*_e_/*σ*, (c) *κ*_L_, (d) *ZT* for Li_2_AgAsX_6_ (X = Cl, Br, I).

The capacity of a material to endure heat is quantified by its specific heat at constant volume (*C*_V_), portrayed against temperature in Fig. 7(a). The *C*_V_ value steadily increases from 0.411, 0.501, and 0.752 J mol^−1^ K^−1^ at 200 K to 0.821, 1.235, and 2.22 J mol^−1^ K^−1^ at 600 K for Li_2_AgAsCl_6_, Li_2_AgAsBr_6_, and Li_2_AgAsI_6_, respectively, underscoring their potential for future-oriented devices.^50^ Understanding the complex interaction of *σ* and *κ*_e_ is facilitated by the thermal-to-electrical conductivity (*κ*_e_/*σ*) ratio, as it is challenging to disentangle the electronic component of *κ*_e_ from the total. The conduction of heat includes both charge carriers and lattice vibration. This ratio can be expressed as a polynomial with coefficients *A*_0_, *A*_1_, *A*_2_, *A*_3_ and *A*_4_:

3 *κ*_e_/*σ* = *A*_0_ + *A*_1_*T* + *A*_2_*T*^2^ + *A*_3_*T*^3^ + *A*_4_*T*^4^

Notably, the *A*_1_ coefficient of the linear term aligns with the Weidman–Franz law^51^*κ*_e_/*σ* = *L*_0_*T* where *L*_0_ = 2.44 × 10^−8^ (a.u.). Hence, the *κ*_e_/*σ* ratio is exceedingly minute, of the order of 10^−5^, enhancing the appeal of these compounds for thermoelectric devices, as demonstrated in Fig. 7(b). To evaluate the contribution of lattice vibration to *κ*_e_, values of *κ*_L_ are calculated and presented in Fig. 7(c). The negligible values of *κ*_L_ for the studied compounds confirm a minimal contribution, auguring well for the superior performance of these materials.^52^ At 200 K, the *κ*_L_ values are noted to be 0.163, 0.147, and 0.17 W m^−1^ K^−1^ for Li_2_AgAsCl_6_, Li_2_AgAsBr_6_, and Li_2_AgAsI_6_, respectively, decreasing to 0.06, 0.04, and 0.02 W m^−1^ K^−1^ at 600 K, highlighting the importance of the studied materials for futuristic thermoelectric devices.^53^ The figure of merit (*ZT*) delineates the efficiency scale of the investigated compositions for practical applications. The computed values, illustrated in Fig. 7(d) against temperature, indicate *ZT* values of 0.43/0.53/0.62 for Li_2_AgAsCl_6_, Li_2_AgAsBr_6_, and Li_2_AgAsI_6_, respectively, at 300 K, diminishing with increasing temperature and reaching 0.27/0.32/0.41 at 600 K. Hence, the investigated compounds exhibit exceptional thermoelectric performance at low and ambient temperatures.

### 3.5. Mechanical properties

The mechanical features of Li_2_AgAsX_6_ (X = Cl, Br, I) were analyzed utilizing the Chapin approach. For cubic symmetry, three elastic constants (*C*_11_, *C*_12_, and *C*_44_) are appropriate to depict mechanical response.^54^ The calculated elastic constant satisfies Born's conditions: *C*_11_ − *C*_12_ > 0, *C*_44_ > 0, *C*_11_ + 2*C*_12_ > 0, and *C*_12_ < *B*_o_ < *C*_11_.^38^ The elastic constants for Li_2_AgAsI_6_ are lower than those for Li_2_AgAsCl_6_ and Li_2_AgAsBr_6_, indicating that Li_2_AgAsCl_6_ and Li_2_AgAsBr_6_ are mechanically more stable than Li_2_AgAsI_6_. The computed bulk (*B*), shear (*G*), and Young's (*Y*) moduli are shown in Table 2, revealing higher values for Li_2_AgAsCl_6_, which suggests greater rigidity. The Pugh (*B*/*G*) ratio is used to differentiate between brittle (Pugh ratio less than 1.75) and ductile (Pugh ratio greater than 1.75) natures.^55^ The values in Table 2 indicate that the investigated compounds exhibit ductile behavior, as verified by the Poisson (*ν*) ratio, *i.e.*, *ν* > 0.26. Comparatively, Li_2_AgAsBr_6_ is more ductile than Li_2_AgAsCl_6_ or Li_2_AgAsI_6_, as reflected in its higher Pugh and Poisson ratios. A value of *A* = 1 indicates the isotropic nature of a material, while deviations indicate anisotropic behavior.^56^ According to the values in Table 2, Li_2_AgAsCl_6_ is more anisotropic than Li_2_AgAsBr_6_ or Li_2_AgAsI_6_. The Navier equations are also scrutinized to determine the average sound velocity (*v*_m_) by separating its transverse and longitudinal components using the equations provided in ref. 57. The Debye temperature (*θ*_D_) is then computed based on *v*_m_ using the formula:4
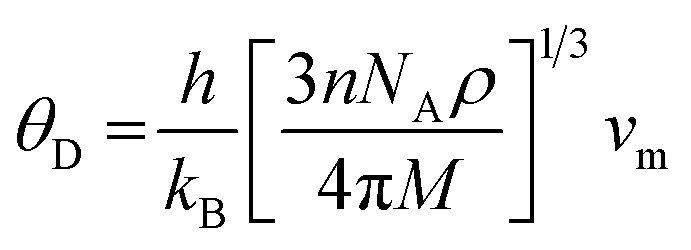
where *ρ*, *M*, and *N*_A_ represent constants with fixed magnitudes. The calculated value of *θ*_D_ is higher for Li_2_AgAsCl_6_ than for Li_2_AgAsBr_6_ or Li_2_AgAsI_6_, indicating that lattice vibrations are more sustainable for Cl-based compounds than for Br- or I-based compounds.^58^ Furthermore, the directional elastic constant *C*_11_ is utilized to estimate the melting temperature (*T*_m_) of the investigated compositions using the equation:

5 *T*_m_ = 553 + 5.91*C*_11_/GPa

**Table 2:** Mechanical and thermodynamic characteristics of Li_2_AgAsX_6_ (X = Cl, Br, I)

Parameter	Li_2_AgAsCl_6_	Li_2_AgAsBr_6_	Li_2_AgAsI_6_
*C*_11_ (GPa)	75.35	50.20	37.96
*C*_12_ (GPa)	26.14	17.66	11.40
*C*_44_ (GPa)	21.00	13.20	8.92
*B* (GPa)	42.67	28.50	20.25
*G* (GPa)	22.44	14.35	10.46
*E* (GPa)	57.29	36.87	26.78
*B*/*G*	1.90	1.98	1.94
*ν*	0.276	0.284	0.279
*A*	0.84	0.81	0.67
*V*_l_ (km s^−1^)	4.93	3.47	2.78
*V*_t_ (km s^−1^)	2.74	1.90	1.54
*V*_m_ (km s^−1^)	3.05	2.12	1.72
*θ*_D_ (K)	267	177	133
*T*_m_ (K)	1000	850	777
*H*_a_ (GPa)	9.44 × 10^6^	6.54 × 10^6^	5.39 × 10^6^
*K*_min_ (W m^−1^ K^−1^)	0.197	0.125	0.088

The calculated *T*_m_ suggests that the investigated compositions can be fabricated at RT (Table 2). The investigation reveals that *T*_m_ is higher for Li_2_AgAsCl_6_ than for Li_2_AgAsBr_6_ or Li_2_AgAsI_6_, suggesting stronger bonding in Li_2_AgAsCl_6_ compared to the rest of the compositions. This is because of the larger ionic radius of Br and I. Moreover, the hardness of the investigated compounds is determined using the expression:

6 *H*_v_ = 0.92(*G*/*B*)^1.137^*G*^0.708^

This hardness value accounts for changes in compound formation and their directional parameters, as described by Tian *et al.*^59^ for a cubic structure. The hardness values are presented in Table 2, confirming that Li_2_AgAsCl_6_ is harder than Li_2_AgAsBr_6_ or Li_2_AgAsI_6_, due to its larger shear modulus and stronger bonding. To understand the lattice dynamics, the minimum lattice conductivity *K*_min_ of the investigated compounds is calculated using the Cahill criterion:^60,61^7
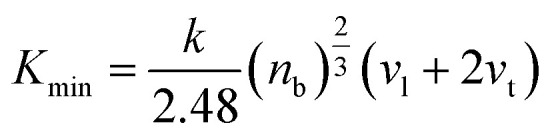


The *K*_min_ values are tabulated in Table 2, indicating very low values for the investigated compounds, in agreement with *κ*_L_ calculated using ShengBTE code. Consequently, the lower *κ*_L_ and higher *ZT* and light energy absorption in the visible-IR range make these materials excellent contenders for renewable energy applications.

### 3.6. Elastic anisotropy

**Fig. 8:**
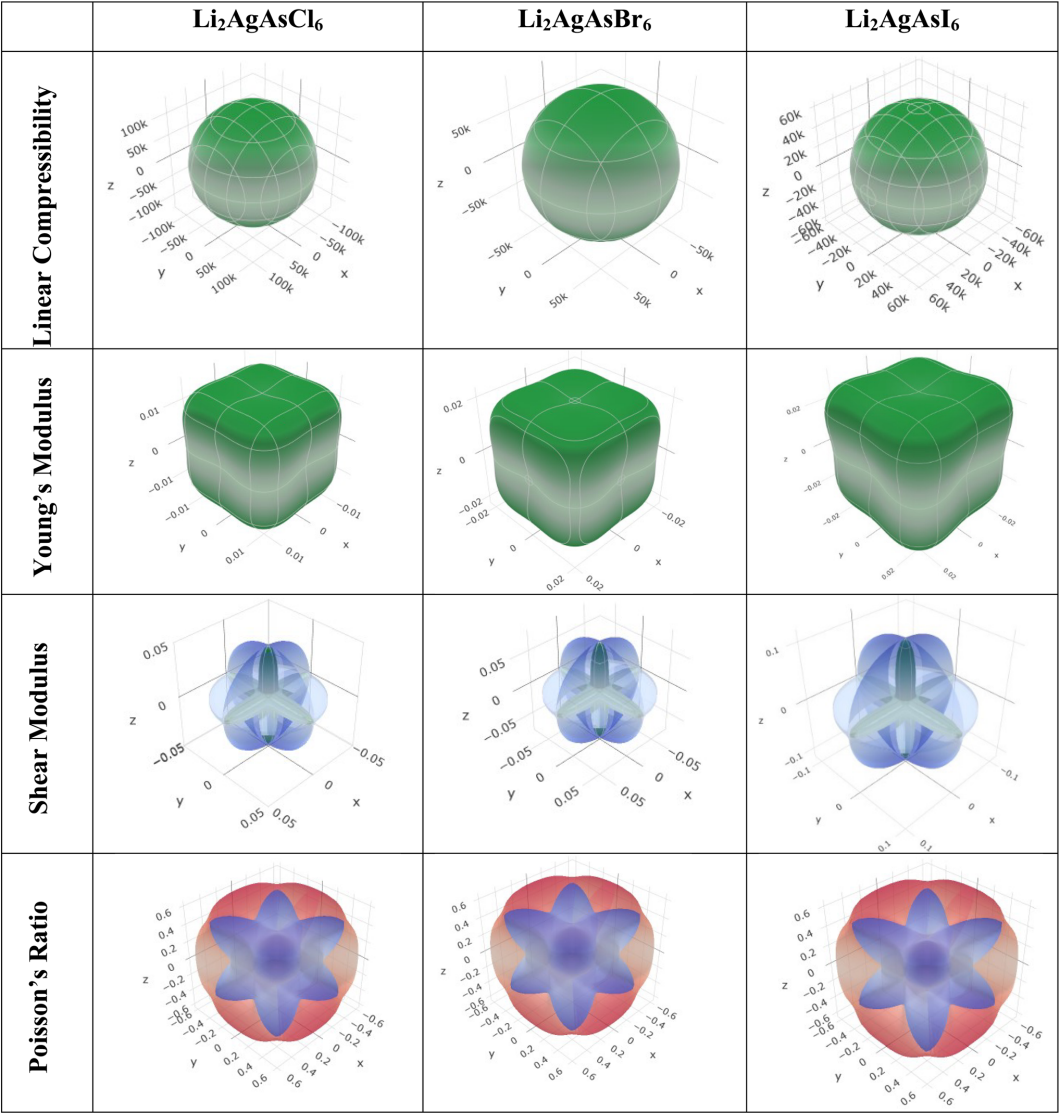
3D representations of elastic modulus for Li_2_AgAsX_6_ (X = Cl, Br, I).

**Fig. 9:**
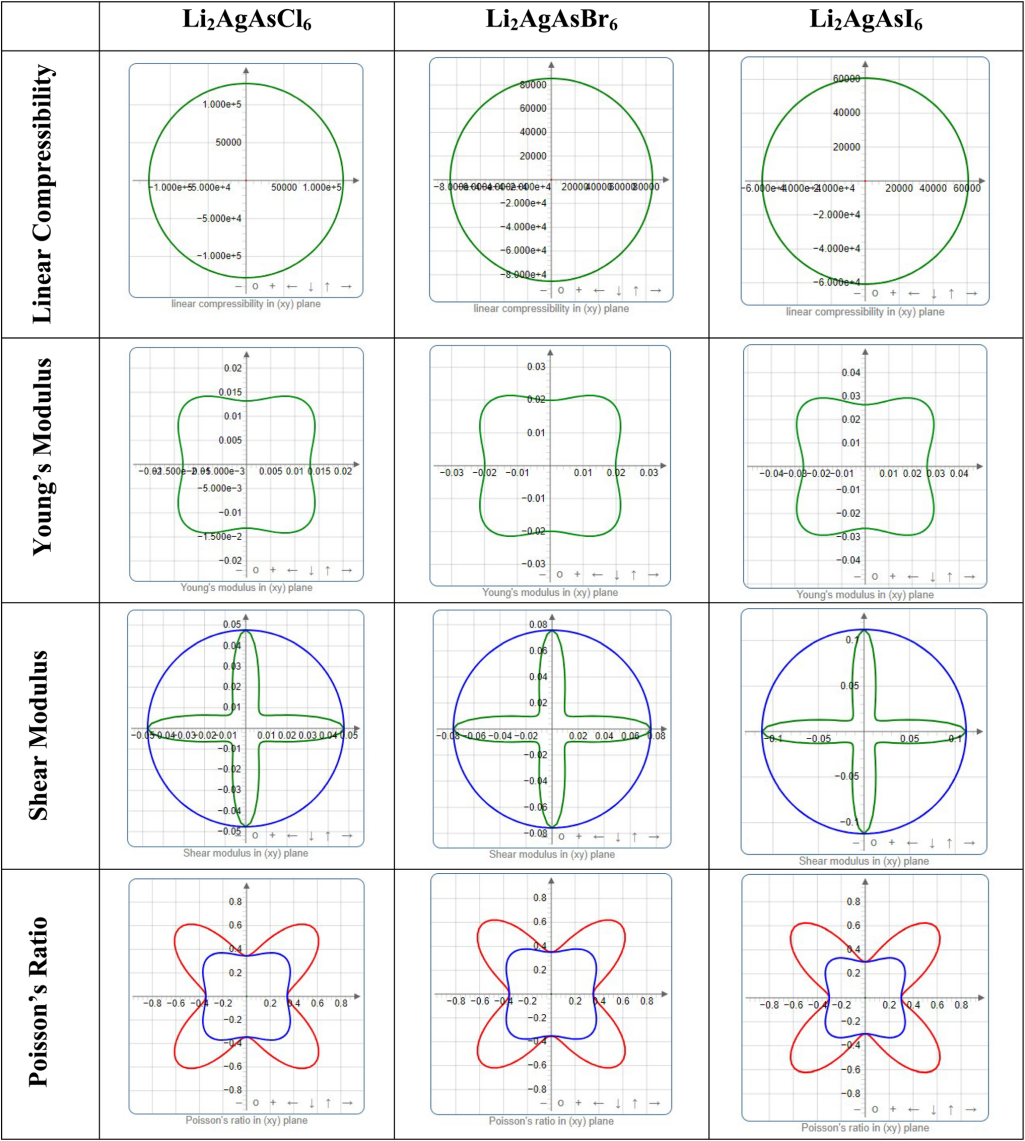
2D plots of elastic modulus for Li_2_AgAsX_6_ (X = Cl, Br, I).

Elastic anisotropy represents a crucial physical attribute of a material. Anisotropic materials demonstrate variations in their chemical and physical properties with directional changes, displaying discrepancies across axes.^62^ In contrast, an isotropic material shows reliable performance irrespective of the direction of measurement. Fig. 8 and 9 depict 3D plots and 2D representations of linear compressibility (*β*), Young's (*Y*) and shear (*G*) moduli, and Poisson's (*ν*) ratio for Li_2_AgAsX_6_ (X = Cl, Br, I).^63^ A spherical 3D surface structure indicates isotropy, while deviations from a sphere indicate anisotropy, with greater deviations indicating stronger anisotropy.^64^ The 3D figures for Li_2_AgAsI_6_ do not resemble spheres, indicating anisotropy. Further insights into the elastic anisotropy of Li_2_AgAsI_6_ are obtained by examining the values of *β*_max_, *β*_min_, *Y*_max_, *Y*_min_, *G*_max_, *G*_min_, *ν*_max_, and *ν*_min_, with (*β*_max_/*β*_min_, *Y*_max_/*Y*_min_, *G*_max_/*G*_min_, and *ν*_max_/*ν*_min_) ratios presented in Table 3. For an isotropic material, these ratios equal 1, while for anisotropic materials, they deviate from 1, with higher ratios indicating stronger anisotropy.^65^ For Li_2_AgAsI_6_, *β*_max_/*β*_min_ = 1, *Y*_max_/*Y*_min_ = 1.6, *G*_max_/*G*_min_ = 5.9, and *ν*_max_/*ν*_min_ = 0.3, indicating greater anisotropy compared to Li_2_AgAsCl_6_ or Li_2_AgAsBr_6_.^66^

**Table 3:** The maximum and minimum values of Young's modulus, linear compressibility, shear modulus and Poisson's ratio with their ratios

Compound	Young's modulus (*Y*) (GPa)			Linear compressibility (*β*)			Shear modulus (*G*) (GPa)			Poisson's ratio (*ν*)		
	*Y*_min_	*Y*_max_	*A*	*β*_min_	*β*_max_	*A*	*G*_min_	*G*_max_	*A*	*ν*_min_	*ν*_max_	*A*
Li_2_AgAsCl_6_	0.013	0.020	1.5	128 030	128 030	1	0.01	0.05	4.7	−0.8	−0.3	0.4
Li_2_AgAsBr_6_	0.019	0.030	1.5	85 520	85 520	1	0.01	0.07	4.9	−0.8	−0.3	0.4
Li_2_AgAsI_6_	0.026	0.43	1.6	60 760	60 760	1	0.02	0.11	5.9	−0.9	−0.3	0.3

## 4. Conclusion

The current report investigates the structural, electronic, optical, thermoelectric, and mechanical characteristics of Li_2_AgAsX_6_ (X = Cl, Br, I) DPs utilizing WIEN2k simulation. We employed the mBJ potential within the FP-LAPW method to ensure precise calculations. The structural and thermodynamic stability of the studied compositions was confirmed by formation enthalpy values ranging from −3.63 to −2.16 eV and tolerance factors between 0.96 and 0.93. The Born's stability conditions also confirmed the structural stability of these compounds. The bandgap decreased from 0.86 eV, to 0.56 eV, and 0.22 eV when Br and I replaced Cl. This bandgap tuning within the infrared region makes Li_2_AgAsX_6_ (X = Cl, Br, I) favorable for infrared photodetector applications and as an alternative to inorganic/organic materials. Moreover, Li_2_AgAsI_6_ exhibits extensive span absorption with negligible optical loss in the visible range; as the temperature increases, *σ* and *κ*_e_ rise linearly, aligning with the observed trend in bandgap. A high S, with a low *κ*_e_/*σ* ratio and *ZT* close to unity, indicate that Li_2_AgAsX_6_ (X = Cl, Br, I) show significant promise for thermoelectric power generation applications.

## Data availability

All data included in this study will be available on request.

## Conflicts of interest

On behalf of all authors, the corresponding author states that there is no conflict of interest.
